# Clinical observations, environmental parameters, and gut microbiota alterations associated with gas bubble disease in the endangered Anji salamander (*Hynobius amjiensis*)

**DOI:** 10.3389/fvets.2026.1900113

**Published:** 2026-08-28

**Authors:** Haiyan Dong, Minglan Dong, Xiaolu Jin, Zhedong Lang, Xianting Wang, Jiabao Xu, Zixing Hu, Xiang Xu

**Affiliations:** 1 Department of Basic Medical Science, Huzhou Normal University, Huzhou, Zhejiang, China; 2 Anji Salamander (Hynobius amjiensis) National Nature Reserve Management Center, Huzhou, Zhejiang, China; 3 National-Local Joint Engineering Laboratory of Aquatic Animal Genetic Breeding and Nutrition, Zhejiang Provincial Key Laboratory of Aquatic Resources Conservation and Development, Key Laboratory of Aquatic Animal Genetic Breeding and Nutrition of Chinese Academy of Fishery Sciences, Huzhou Normal University, Huzhou, Zhejiang, China

**Keywords:** ammonia nitrogen, gas bubble disease, gut microbiota, *Hynobius amjiensis*, total dissolved gas supersaturation

## Abstract

Gas bubble disease (GBD) is a non-infectious disease caused by the supersaturation of total dissolved gas (TDG) in aquatic environments, which can damage multiple tissues and organs in aquatic animals and be potentially fatal in severe cases. This study reports, for the first time, clinical symptoms resembling GBD in wild larvae of the endangered Anji salamander (*Hynobius amjiensis*). By combining clinical observations, habitat water quality testing, and 16S rRNA sequencing, we investigated the ecological drivers of this disease and its impact on the host’s fecal microbial community. Affected individuals exhibited severe subcutaneous and abdominal emphysema, buoyancy abnormalities, and anorexia; these symptoms resolved spontaneously within 24–72 h after transfer to stable water bodies. Puddles where cases occurred exhibited severe eutrophication and massive algal blooms, leading to extreme TDG supersaturation, primarily driven by dissolved oxygen. Furthermore, the species evenness of the fecal microbiota in the affected group showed a decreasing trend, with a massive loss of core commensal bacteria, while the relative abundance of the opportunistic pathogen *Aeromonas* increased abnormally. These findings indicate that eutrophication-driven physicochemical abnormalities in water bodies may be the primary environmental stressors inducing GBD, while the disease itself may disrupt the organism’s homeostasis, further triggering secondary dysbiosis of the gut microbiota. This study provides a reference for understanding the ecologically driven mechanisms of non-infectious diseases in wild amphibians, as well as for the management and conservation of aquatic environments in the habitats of endangered amphibian species.

## 1 Introduction

Gas bubble disease (GBD) is a non-infectious disease caused by the supersaturation of total dissolved gas (TDG) in aquatic environments and has been widely reported in aquatic animals (1). GBD typically occurs when dissolved gas concentrations, particularly oxygen and nitrogen, exceed equilibrium levels relative to ambient atmospheric pressure, and it is especially common in water bodies with limited water exchange, such as springs and groundwater systems (1, 2). The pathological mechanism of GBD is analogous to decompression sickness in mammals, including humans and cetaceans, in which abrupt environmental pressure changes cause dissolved gases to come out of solution and form intravascular or tissue bubbles, resulting in vascular obstruction, tissue compression, and physiological dysfunction (3–5). This mechanism is also recognized as the key pathological basis for GBD in aquatic animals. In fish, susceptibility to GBD varies among species and developmental stages, with juveniles generally being the most sensitive. Depending on the degree of TDG supersaturation, GBD may occur in either a chronic form (approximately 103% TDG saturation) or an acute form (>110–115% TDG saturation), and severe cases may result in high mortality (1, 6–8). In recent years, increasing attention has been directed toward the occurrence of GBD in amphibians, with confirmed cases reported in species such as *Xenopus laevis* (9) and *Andrias davidianus* (10). However, most studies of amphibian GBD have been restricted to laboratory or captive conditions, whereas reports from natural breeding habitats of endangered wild amphibians remain exceedingly scarce.

Amphibian larvae are highly dependent on aquatic environments, and their exposed, permeable skin and highly vascularized external gills render them particularly sensitive to fluctuations in dissolved gas concentrations (11, 12). Alterations in aquatic environmental conditions may not only directly induce GBD but also indirectly influence host health through changes in microbial community composition. Recent studies have demonstrated that microbial communities in aquatic environments are closely associated with the health status of aquatic animals, and microbial imbalance may increase the relative abundance of opportunistic pathogens, thereby disrupting host physiological homeostasis (13). Consequently, exploring the mechanisms of disease onset from the dual perspectives of environmental factors and microbial communities contributes to a more comprehensive understanding of the ecological background of aquatic animal diseases.

Previous studies have shown that GBD can lead to structural imbalances in the gut microbiota of aquatic animals. For example, in *Pampus argenteus*, intestinal bubble accumulation resulted in reduced species richness and evenness of the gut microbiota, as well as a marked increase in the relative abundance of aerobic bacteria (14). In bullfrog tadpoles, intestinal bubble accumulation altered the anaerobic microenvironment, resulting in microbial dysbiosis characterized by a decline in beneficial bacteria and an increase in opportunistic pathogens. This process subsequently affected liver health through the gut–liver axis, forming a pathological cycle involving intestinal injury, microbial dysbiosis, and hepatic oxidative stress (15). Furthermore, secondary infections caused by the opportunistic pathogen *Vibrio splendidus*, accompanied by inflammatory cell infiltration, have been reported at GBD lesion sites in syngnathid fishes (*Syngnathus schlegeli* and *Hippocampus haema*) (16). These findings suggest that tissue damage and barrier disruption induced by GBD may facilitate colonization by environmental opportunistic pathogens, thereby triggering secondary intestinal dysbiosis. Nevertheless, studies examining the relationship between GBD and gut microbiota dynamics in wild, endangered amphibians remain scarce.

*Hynobius amjiensis* is a critically endangered (CR) species endemic to China and belongs to the class Amphibia, order Caudata, family Hynobiidae, and genus *Hynobius*. Currently, the species is restricted to high-altitude *Sphagnum* peat bog habitats at elevations ranging from approximately 1,300 to 1,600 m, including Longwang Mountain in Anji, Qingliang Peak in Lin’an (Zhejiang Province), and Qingliang Peak in Anhui Province (17, 18). Adult *H. amjiensis* typically remain concealed within humus layers beneath sphagnum moss and enter shallow natural pools only during the breeding season, from November to March, for courtship and spawning (19). Following hatching, larvae adopt a fully aquatic lifestyle and undergo prolonged developmental stages, including embryonic development, balancer development, and limb development, before metamorphosis and transition to terrestrial habitats. Owing to factors such as cannibalism, the proportion of larvae successfully reaching metamorphosis is generally less than 1% of hatched individuals (20–22). This strong dependence on aquatic habitats renders the species highly vulnerable to ecological risks associated with fluctuations in water quality. Natural shallow water pools within the breeding habitats of *H. amjiensis* are characterized by limited water volume and poor water exchange, making them particularly susceptible to seasonal climatic variation and nutrient enrichment, which may induce complex physicochemical abnormalities and environmental stress (23). In the present study, we report for the first time multiple wild *H. amjiensis* larvae exhibiting clinical manifestations highly consistent with GBD. We described the clinical symptoms and recovery process of affected individuals, measured key physicochemical parameters in both the affected and control pools, and employed 16S rRNA high-throughput sequencing to compare fecal microbial community structures between diseased and healthy groups. By integrating clinical characteristics, environmental physicochemical factors, and gut microbial communities, this study aimed to present the phenomenon of GBD in wild *H. amjiensis* larvae and preliminarily explore its causative ecological drivers. Ultimately, these findings are expected to provide a scientific basis for targeted conservation measures to improve larval survival rates, while simultaneously enriching the research data on non-infectious diseases in wild amphibians.

## 2 Materials and methods

### 2.1 Study area

**Figure 1:**
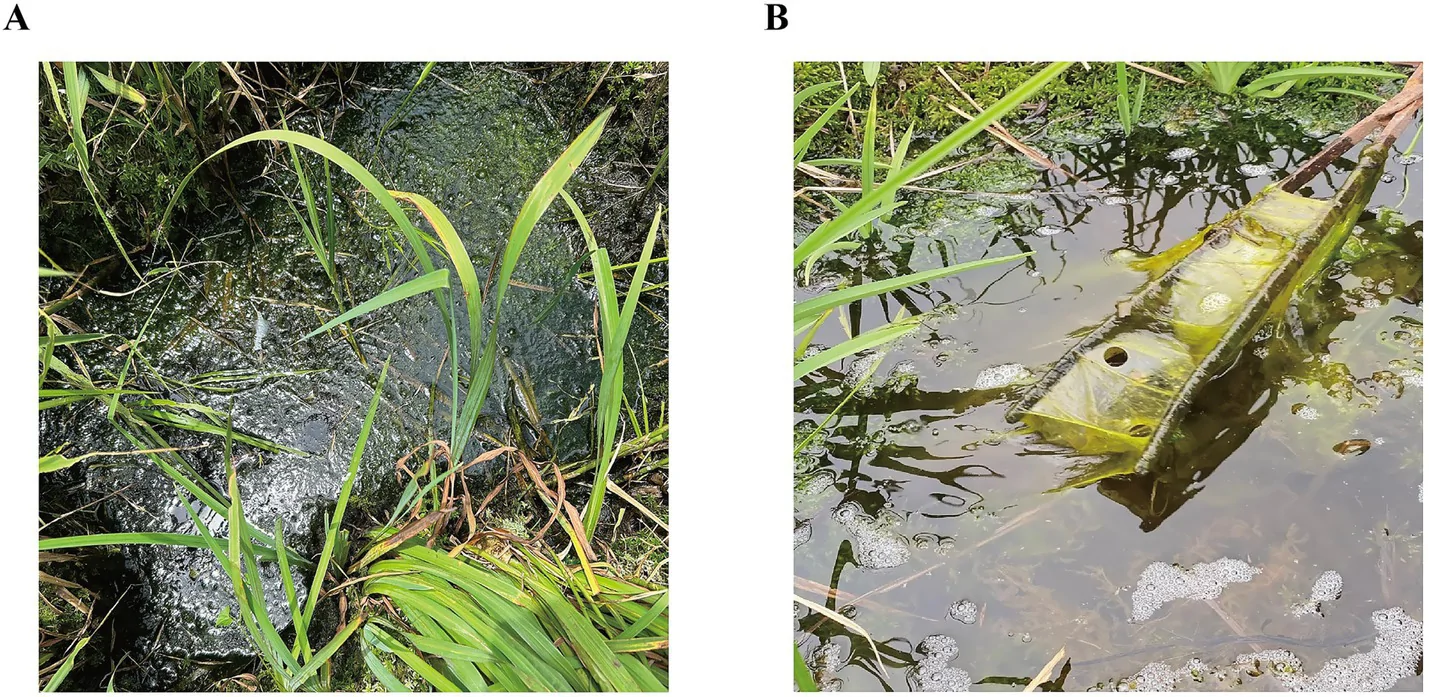
Breeding puddles in the habitat of *Hynobius amjiensis*. **(A)** The breeding puddle where cases of gas bubble disease were found (GBD group). **(B)** The adjacent breeding puddle with healthy individuals (CON group).

Water quality surveys were conducted from May 2023 to May 2025 at the *H. amjiensis* National Nature Reserve on Longwang Mountain in Zhejiang Province (30°29′58″N, 119°26′27″E). The study area is located in the alpine *Sphagnum* bog wetland within the reserve’s core zone, at an elevation of approximately 1,350 m. During a habitat water quality assessment conducted on May 3, 2024, four wild *H. amjiensis* larvae exhibiting suspected GBD were incidentally discovered in one of the natural shallow pools, while no abnormalities were observed in the larvae in adjacent pools. Consequently, the pool where the GBD cases were discovered was designated as the GBD group (Figure 1A), and the adjacent pool with no observed abnormalities was designated as the healthy control group (CON group) (Figure 1B).

### 2.2 Case descriptions, clinical observations, and recovery

Based on macroscopic descriptive clinical observations, bubble distribution, buoyancy status, locomotor activity, and feeding behavior in the affected individuals were qualitatively assessed and recorded. Upon identifying individuals suspected of having GBD, larvae from both the GBD and CON groups were immediately and synchronously transferred to temporary holding containers with adequate aeration and dissolved gas equilibrium (dissolved oxygen maintained at approximately 7.7 mg/L and water temperature at 15 °C), and a 7-day feeding and observation experiment was conducted. During the period when visible bubbles were still present in the GBD-affected larvae, no feeding was provided. After bubble dissipation, sterilized fish flesh was offered to promote larval recovery and reduce the risk of pathogen introduction from exogenous live prey. The feed was prepared as follows: fresh grass carp flesh was autoclaved for 15 min, deboned, cut into small pieces, and placed in glass Petri dishes at the bottom of the holding containers, allowing the larvae to feed voluntarily. The time required for bubble dissipation and the progress of behavioral recovery were recorded to assess the reversibility of the condition.

### 2.3 Environmental and water physicochemical parameters

To investigate the potential environmental triggers of GBD, this study measured the physicochemical parameters of the water in both the GBD group and CON group pools. A portable multi-parameter water quality analyzer (HD-BWY, Haldor) was used to measure nine parameters *in situ*: pH, dissolved oxygen (DO), ammonia nitrogen, phosphate, zinc, total iron, copper, hexavalent chromium, and residual chlorine. Six sampling sites were randomly selected from each pool, with three replicates per site. For each measurement, a 10 mL water sample was collected, gently mixed with the corresponding test reagent, and allowed to stand for 8 min before analysis. During this procedure, strict precautions were taken to minimize water disturbance to prevent the outgassing of dissolved oxygen.

### 2.4 Sample collection

To assess the impact of GBD on the host gut microecology, fecal samples were collected from individuals in the GBD and CON group during the temporary holding period. A total of four larvae showing suspected GBD were collected during the field survey, and each individual was maintained separately as an independent sampling unit. However, DNA extraction failed for one individual because the amount of fecal material obtained was insufficient, and this individual did not produce an adequate amount of additional feces during the 7-day holding period. Therefore, this sample was excluded from the gut microbial community analysis. Because the suspected GBD cases were incidentally discovered during the field survey, the number of available affected larvae was limited, and only a small amount of fecal material could be obtained from each individual. To obtain sufficient material for DNA extraction and sequencing, feces excreted by the same individual during the period when visible gas bubbles were present and the first feces excreted after bubble dissipation and feeding resumption were combined into one sequencing sample. Fecal samples from different individuals were not pooled. Finally, three fecal samples from the GBD group and three fecal samples from the CON group were successfully collected and used for gut microbial community analysis.

### 2.5 16S rRNA sequencing

The collected fecal samples were rapidly frozen in liquid nitrogen, stored on dry ice, and shipped to Majorbio Bio-Pharm Technology Co., Ltd. (Shanghai, China) for 16S rRNA high-throughput sequencing. Total genomic DNA was extracted using the E. Z. N. A.^®^ Stool DNA Kit (Omega Bio-tek, Norcross, GA, United States). The DNA quality and concentration were determined using 1.0% agarose gel electrophoresis and a NanoDrop 2000 spectrophotometer (Thermo Scientific, United States). The V3-V4 hypervariable region of the 16S rRNA gene was amplified using the forward primer 338F (5’-ACTCCTACGGGAGGCAGCAG-3′) and the reverse primer 806R (5’-GGACTACHVGGGTWTCTAAT-3′) on a T100 Thermal Cycler (BIO-RAD, United States). The PCR mixture was including 10 μL of 2 × Pro Taq, 0.8 μL of each primer (5 μM), template DNA (10 ng/μL), and ddH_2_O to a final volume of 20 μL. The PCR amplification program consisted of an initial denaturation at 95 °C for 3 min, followed by 27 cycles of denaturation at 95 °C for 30 s, annealing at 53 °C for 30 s, and extension at 72 °C for 45 s, with a final extension at 72 °C for 10 min and holding at 10 °C. The PCR products were extracted from a 2% agarose gel, purified using the PCR Clean-Up Kit (YuHua, Shanghai, China), and quantified using a Qubit 4.0 fluorometer (Thermo Fisher Scientific, United States). The sequencing library was constructed using the NEXTFLEX Rapid DNA-Seq Kit, and sequencing was performed on an Illumina NextSeq 2000 platform (Illumina, San Diego, CA, United States).

### 2.6 Bioinformatics and statistical analysis

Raw paired-end sequencing reads were quality-filtered using fastp (https://github.com/OpenGene/fastp, version 0.23.4) (24) and subsequently merged using FLASH (http://www.cbcb.umd.edu/software/flash, version 1.2.11) (25). Paired-end reads were merged with a minimum overlap length of 10 bp and a maximum mismatch ratio of 0.2 within the overlap region. The quality-filtered and merged sequences were clustered into operational taxonomic units (OTUs) at 97% sequence identity using USEARCH (26, 27) (http://drive5.com/usearch/, version 11), and chimeric sequences were removed during OTU construction. Representative OTU sequences were taxonomically annotated using the RDP Classifier (28) (http://rdp.cme.msu.edu/, version 2.11) against the SILVA 138.2 16S bacterial database, with a confidence threshold of 70%. After quality control, chimera removal, taxonomic filtering, and rarefaction, the retained OTUs were used for subsequent analyses of the gut microbial community.

Bioinformatic analysis of the gut microbiota was carried out using the Majorbio Cloud platform (https://cloud.majorbio.com) Alpha diversity was assessed using the Chao1 richness, Shannon, and Simpson indices calculated via Mothur (version 1.30.1) (29). The Wilcoxon rank-sum test was used to evaluate intergroup differences in alpha diversity. Beta diversity was measured using the Bray-Curtis distance. Principal Coordinate Analysis (PCoA) was used to examine the similarity of community structures among the samples. The Linear Discriminant Analysis Effect Size (LEfSe) algorithm (set with LDA score > 2.0 and *p* < 0.05) was employed to identify bacterial taxa exhibiting significant differences in abundance from the phylum to genus levels between the groups (30).

To evaluate the differences in water physicochemical parameters between the GBD and CON groups, the three technical replicates at each sampling point were averaged to yield a representative value (*n* = 6 per group). Statistical differences between groups were analyzed using the Mann–Whitney U test. Significant differences were defined as *p* ≤ 0.05 and as a trend if 0.05 < *p* ≤ 0.10. All statistical analyses were performed using GraphPad Prism 10.

## 3 Results

### 3.1 Pathological description, clinical observations, and recovery from gas bubble disease in *H. amjiensis*

**Figure 2:**
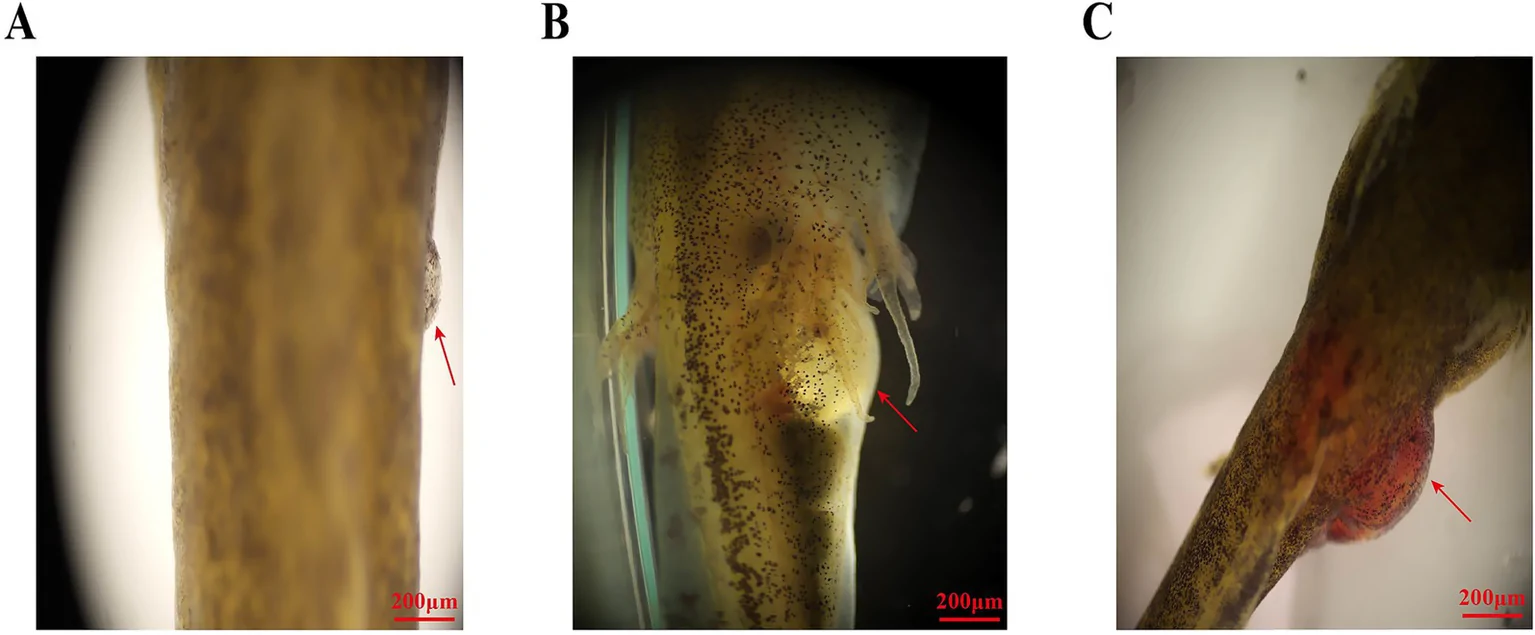
Macroscopic pathological features of gas bubble disease in *Hynobius amjiensis*. **(A)** Distinct gas bubbles observed in the abdomen (red arrow). **(B)** Gas bubble formation within the subcutaneous tissues of the trunk (red arrow). **(C)** A large hyperemic gas bubble formed locally in a severely affected individual, leading to prominent swelling of the trunk (red arrow). Scale bars: 200 μm.

Macroscopically, distinct gas bubbles were observed on the body surface and within the body cavities of affected individuals, primarily distributed in the head (Supplementary Figure S1), abdominal cavity (Figure 2A), and subcutaneous tissues of the trunk (Figure 2B). In severely affected individuals, large gas bubbles accompanied by severe hyperemia or hemorrhage formed within the abdominal cavity, causing localized abnormal swelling of the trunk (Figure 2C). Behaviorally, compared to healthy individuals, affected *H. amjiensis* larvae generally exhibited significant locomotor impairments and buoyancy abnormalities. Specifically, they displayed impaired diving ability, a tendency to lose equilibrium, and sluggish swimming; a large number of affected individuals floated at the water’s surface for extended periods or passively rested on algal mats. Additionally, a marked reduction in feeding behavior was observed in affected individuals, with severely affected individuals even exhibiting severe inappetence or complete anorexia.

In this study, affected individuals were transferred to a temporary holding container with water at normal dissolved oxygen saturation levels and stable water quality for observation. The results indicated that the clinical signs of GBD are reversible in the short term. In most individuals, subcutaneous and intracoelomic bubbles dissipated spontaneously after removal from the supersaturated *in situ* pools; among them, individuals with mild symptoms exhibited complete bubble disappearance within 24 h and resumed normal activity, while those with larger bubbles experienced natural dissipation within 48–72 h and gradually resumed normal swimming, diving, and feeding behaviors (Supplementary Table S1).

### 3.2 Environmental parameters

**Figure 3:**
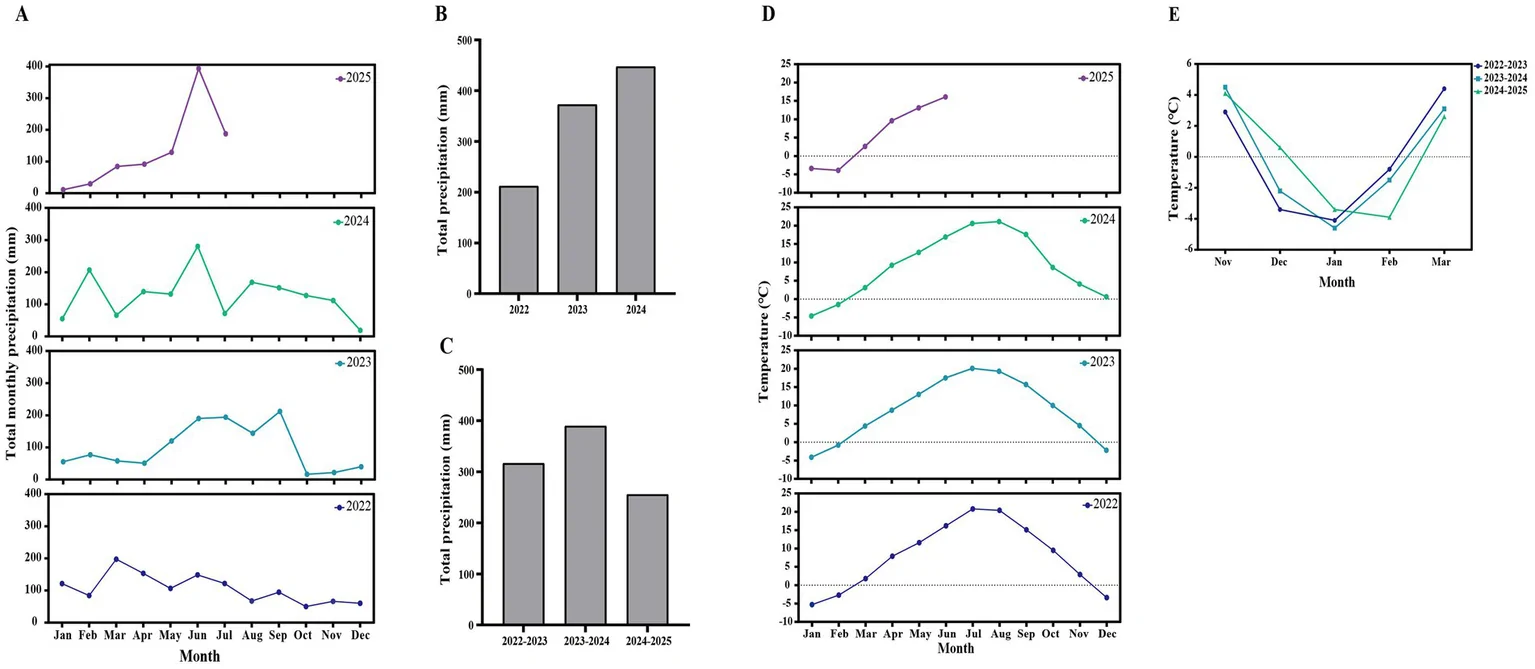
Temperature and precipitation variations in the breeding habitat of *Hynobius amjiensis* from 2022 to 2025. **(A)** Mean monthly precipitation from January 2022 to July 2025. **(B)** Total precipitation during the autumn period for water storage (August–October) in 2022, 2023, and 2024. **(C)** Total precipitation during the breeding season (November–March) from 2022 to 2025. **(D)** Mean monthly temperature from January 2022 to July 2025. **(E)** Temperature variations during the breeding season (November–March) from 2022 to 2025.

To investigate the macro-environmental drivers of GBD, this study analyzed continuous meteorological monitoring data from the habitat of wild *H. amjiensis* from 2022 to 2025. The results indicated that air temperature and precipitation this breeding habitat exhibited distinct seasonal and interannual fluctuations, which could profoundly influence the hydrological conditions of the shallow breeding pools within the habitat. Precipitation exhibited an uneven distribution across years and seasons (Figure 3A). During the autumn water accumulation period (August–October), total precipitation showed an annual upward trend, increasing from 212.2 mm in 2022 to 447.8 mm in 2024 (Figure 3B). However, during the critical stage of embryonic development (November to March of the following year), precipitation fluctuated dramatically, with cumulative precipitation from November 2024 to March 2025 dropping sharply to 255.9 mm (Figure 3C). Similarly, monthly average temperatures exhibited a typical seasonal rhythm (Figure 3D). Notably, during the critical breeding and early larval development period of *H. amjiensis* (November to March of the following year), winter extreme low temperatures in the recent two cycles (2023–2025) were milder compared to the 2022–2023 period (Figure 3E). Temperatures rose rapidly in spring (April–May), providing favorable thermal conditions for the proliferation of primary producers, such as benthic algae.

Field observations revealed marked seasonal environmental variation and potential habitat stressors in the natural breeding habitat of *H. amjiensis* (Supplementary Figure S2). In spring, dense vegetation was observed around the breeding pools, with high coverage of weeds and other plants in some areas. These plants appeared to compete spatially with native *Sphagnum* moss, a key breeding substrate, potentially contributing to the shrinkage and degradation of the *Sphagnum*-dominated microhabitat on which *H. amjiensis* depends (Supplementary Figures S2A, S2B). Under low-temperature winter conditions, the breeding pools of *H. amjiensis* were frozen, whereas after spring warming and ice melt, some pools still exhibited low water levels (Supplementary Figures S2C, S2D). In addition, organic matter accumulation and apparent signs of water quality deterioration were observed in some breeding pools during spring (Supplementary Figure S2E). These observations suggest that *H. amjiensis* larvae may be exposed to multiple environmental stressors during early development, including water scarcity, habitat degradation, and deteriorating water conditions.

To further assess the water quality triggers inducing GBD, this study measured physicochemical parameters in pools with occurrences of GBD and dense algal growth (GBD group) and in adjacent healthy pools with sparse algal growth (CON group). The results (Table 1) showed that, under consistent water temperatures (17.8 ± 0.4 °C) during the sampling period, there were significant differences in pH, dissolved oxygen, ammonia nitrogen, and phosphate concentrations between the two groups of pools (*p* < 0.05). The pH value of the GBD group (5.076 ± 0.058) was distinctly acidic, its dissolved oxygen concentration was as high as 8.397 ± 0.177 mg/L, and the concentrations of phosphate (1.805 ± 0.003 mg/L) and ammonia nitrogen (0.3093 ± 0.0377 mg/L) were both higher than normal baseline levels. In contrast, the control group’s water bodies exhibited a slightly acidic pH (6.633 ± 0.058), and it’s dissolved oxygen concentration (7.623 ± 0.121 mg/L), phosphate (0.381 ± 0.058 mg/L), and ammonia nitrogen levels (0.002 ± 0.001 mg/L) were relatively lower and within normal ranges.

**Table 1:** Physicochemical water quality parameters of breeding pools in the gas bubble disease (GBD) and control (CON) groups.

Water parameter	GBD	CON	Reference value
pH	5.076 ± 0.058*	6.633 ± 0.058	7.0 ~ 8.2
DO(mg/L)	8.397 ± 0.177*	7.623 ± 0.121	6 ~ 8
Ammonia Nitrogen(mg/L)	0.3093 ± 0.0377*	0.002 ± 0.001	0.01 ~ 0.2
Phosphate(mg/L)	1.805 ± 0.003*	0.381 ± 0.058	0.01 ~ 0.1
Zinc(mg/L)	0.043 ± 0.046	0.043 ± 0.005	0.05 ~ 3
Total iron(mg/L)	ND	ND	0.02 ~ 5
Copper(mg/L)	ND	ND	0.1 ~ 5
ChromiumVI(mg/L)	0.012 ± 0.002	0.012 ± 0.0015	0.005 ~ 1
Residual chlorine(mg/L)	ND	ND	<0.01

GBD, breeding pools with gas bubble disease cases; CON, adjacent breeding pools with healthy individuals; DO, dissolved oxygen. Values are presented as mean ± standard deviation (SD). ND indicates not detected, below the detection limit of the analytical method. Statistical differences between groups were analyzed using the Mann–Whitney *U* test. * indicates significant differences between groups (*p* < 0.05).

### 3.3 Gut microbiome analysis

Given the physical emphysema and reduced feeding activity observed in GBD-affected larvae, fecal samples collected from the control (CON) and GBD groups were subjected to 16S rRNA sequencing. For the six fecal samples analyzed in this study, a total of 335,291 raw single-end reads were generated. After paired-end read merging, quality control, and chimera removal, 314,474 high-quality merged sequences were retained, with an average of 52,412 ± 11,464 sequences per sample. The average length of the processed sequences was 414 bp. Based on a 97% sequence identity threshold, a total of 966 OTUs were obtained (Supplementary Tables S2, S3), providing sufficient sequencing depth for subsequent gut microbiota analysis.

**Figure 4:**
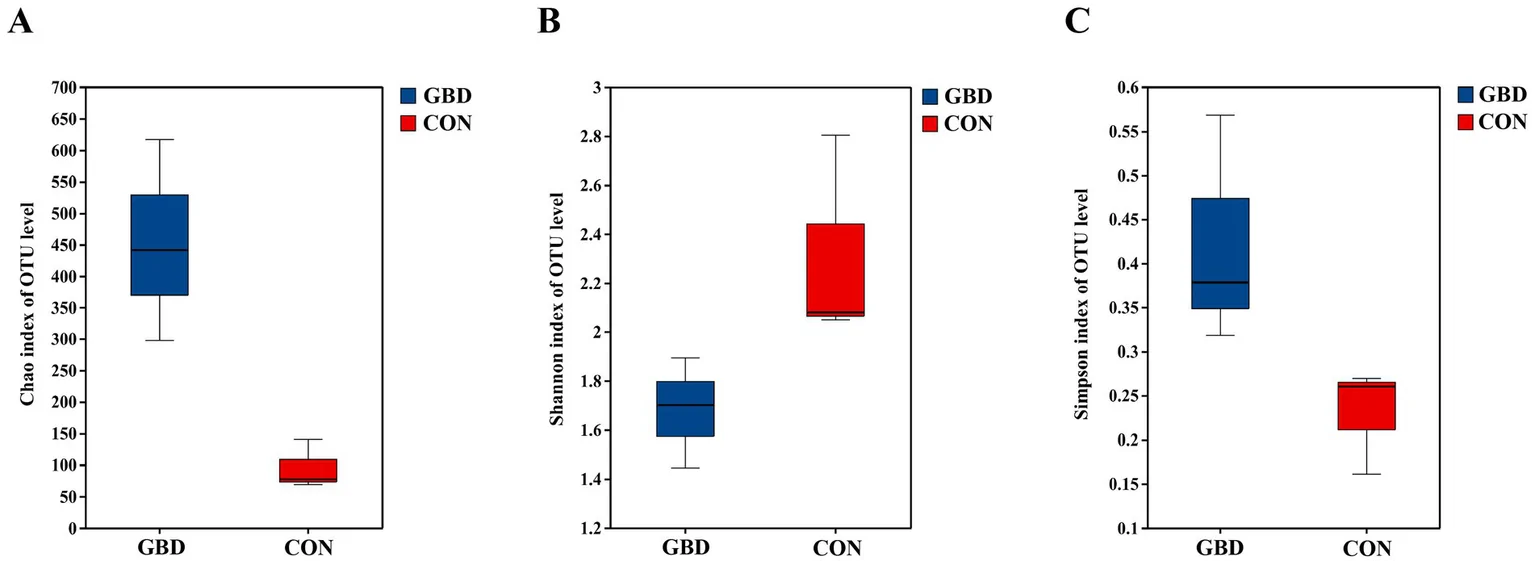
Alpha diversity of the gut microbiota in *Hynobius amjiensis* from gas bubble disease and healthy control groups. **(A)** Chao index, reflecting community richness. **(B)** Shannon index and **(C)** Simpson index, evaluating community diversity. GBD, gas bubble disease group; CON, healthy control group.

Based on the resulting OTU dataset, we further compared the diversity and community structure of the gut microbiota between the CON and GBD groups of *H. amjiensis* larvae. Results of the alpha diversity analysis (Figure 4) revealed no statistically significant differences in microbial community diversity between the two groups. However, numerically, the Chao1 index was higher in the GBD group than in the CON group (Figure 4A), indicating an increase in species richness in the gut microbiota of the GBD group. Conversely, the Shannon index was lower in the GBD group than in the CON group (Figure 4B), while the Simpson index was relatively higher (Figure 4C), which may reflect a decreasing trend in microbial community evenness in the GBD group.

**Figure 5:**
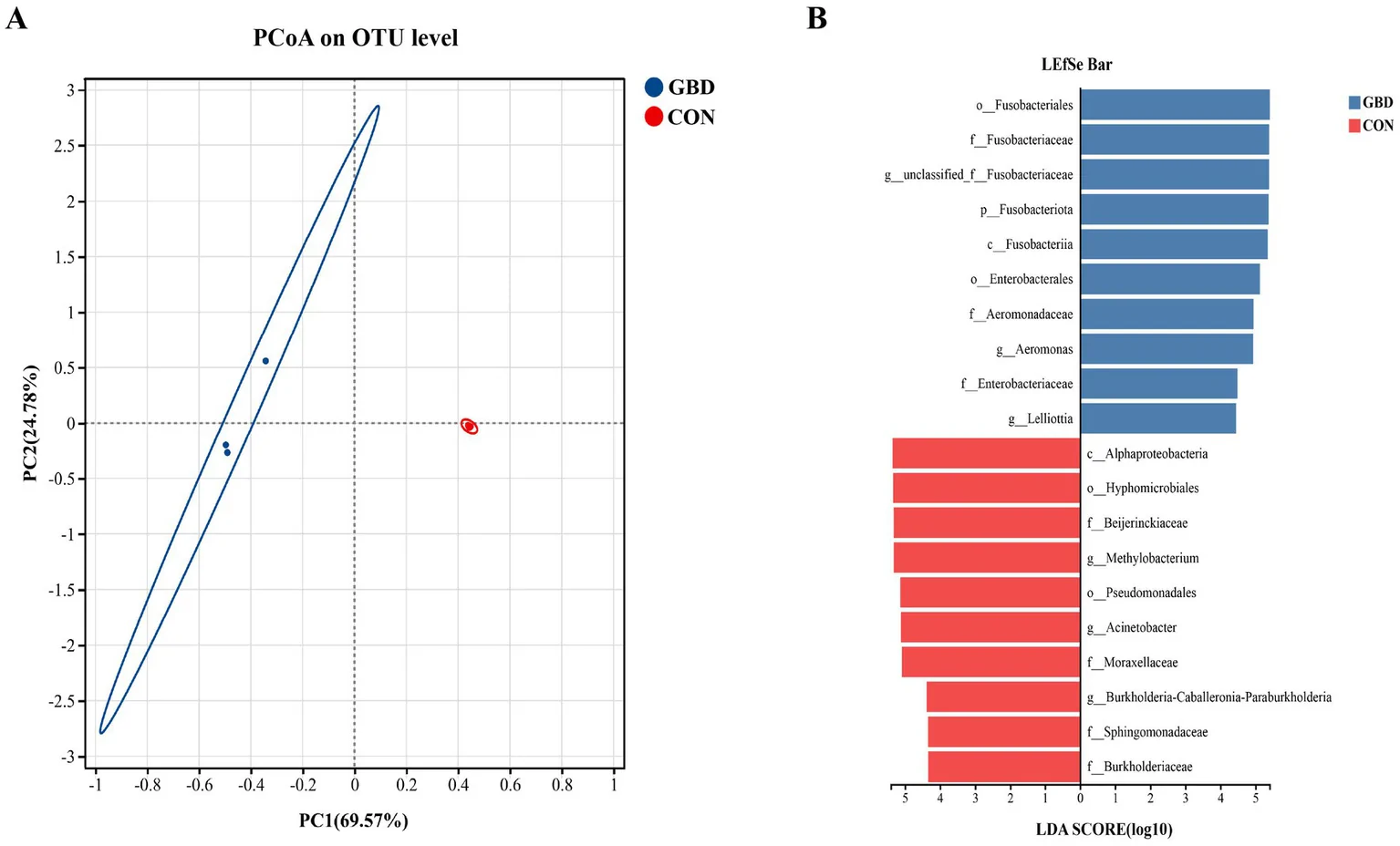
Beta diversity and differential abundance analysis of the gut microbiota in *Hynobius amjiensis* between GBD and CON groups. **(A)** Principal coordinate analysis (PCoA) at the OTU level (*R* = 1, *p* = 0.103). **(B)** Linear discriminant analysis effect size (LEfSe) illustrating biomarker bacterial taxa with significantly differential abundances between the two groups from the phylum to genus levels (LDA score >2, *p* < 0.05). GBD, gas bubble disease group; CON, healthy control group.

The results of Principal Coordinate Analysis (PCoA) (Figure 5A) revealed differences in the beta diversity of the gut microbiota of *H. amjiensis* larvae between the GBD and CON groups. PC1 and PC2 accounted for 69.57 and 24.78% of the total variance, respectively, revealing that the two groups of samples were distributed in distinct spatial regions. LEfSe analysis (LDA > 2, *p* < 0.05) identified a total of 20 differentially abundant taxa, spanning taxonomic levels from phylum to genus (Figure 5B). Among these, taxa such as the order Fusobacteriales, the family Enterobacteriaceae, and the genus *Aeromonas* were significantly enriched in the GBD group, whereas taxa such as the class Alphaproteobacteria, the genus *Acinetobacter*, and the family Burkholderiaceae were significantly enriched in the CON group.

**Figure 6:**
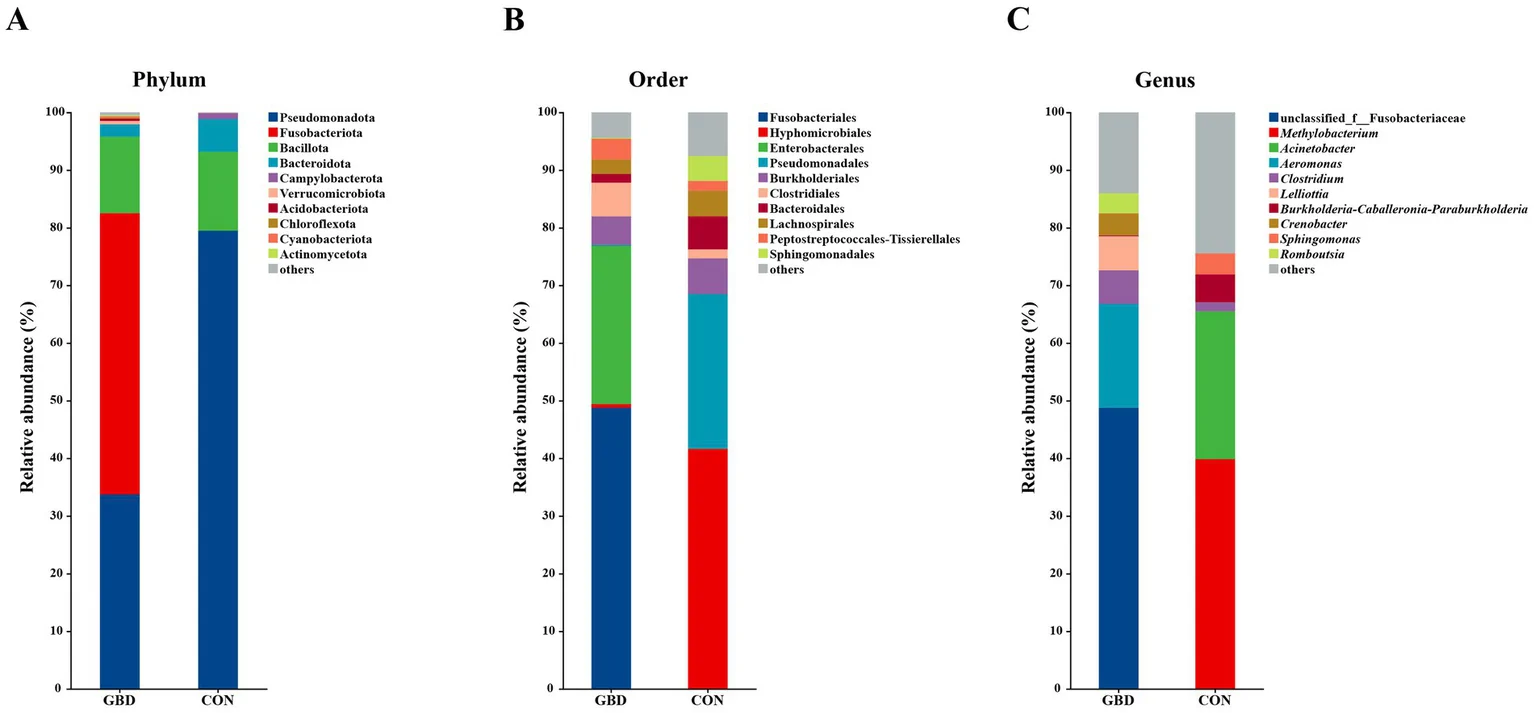
Community composition of the gut microbiota in *Hynobius amjiensis* from gas bubble disease and healthy control groups. Relative abundances of the gut microbial communities between the two groups are shown at the **(A)** phylum, **(B)** order, and **(C)** genus levels. At each taxonomic level, only the top 10 taxa with the highest mean relative abundance are displayed, and the remaining low-abundance taxa are grouped into “Others.” GBD, gas bubble disease group; CON, healthy control group.

In terms of community composition, substantial differences were observed across different taxonomic levels (Figure 6). At the phylum level (Figure 6A), the CON group was dominated by Pseudomonadota (79.48%). However, in the GBD group, the relative abundance of Pseudomonadota (33.76%) decreased, while that of Fusobacteriota (48.77%) substantially increased and became the dominant phylum. At the order level (Figure 6B), the core taxa in the CON group were primarily Hyphomicrobiales (41.63%) and Pseudomonadales (26.84%). In the GBD group, these taxa were largely replaced by Fusobacteriales (48.77%) and Enterobacterales (27.42%). At the genus level (Figure 6C), the dominant genera in the GBD group were primarily unclassified_f_Fusobacteriaceae (48.77%) and *Aeromonas* (17.99%), whereas in the CON group, the relative abundances of *Methylobacterium* (39.87%) and *Acinetobacter* (25.63%) were higher.

## 4 Discussion

### 4.1 Clinical characteristics of gas bubble disease in *H. amjiensis* compared to other aquatic animals

A comparison of the clinical manifestations in *H. amjiensis* with those in other aquatic animals reveals that its symptoms closely resemble those of GBD in fish and other amphibians. In fish, typical symptoms of acute GBD include the accumulation of gas bubbles on the body surface and fins, localized hemorrhaging, and behavioral abnormalities such as sluggish swimming, buoyancy disorders, and abnormal escape behavior (31, 32). When bubbles accumulate in the head or eyes, they can cause ocular swelling or even exophthalmos (33). Although adult fish generally have a higher tolerance for total dissolved gas supersaturation than juveniles, gas embolisms forming in gill filaments or muscle tissue can still significantly impair their locomotor ability and cause physiological dysfunction (8, 34). Among amphibians, GBD in *Xenopus laevis* often manifests as buoyancy abnormalities and punctate or patchy hemorrhages on the skin of the webbing and legs. In the early stages of the disease, these lesions are mostly concentrated on the hind limbs and ventral side, subsequently spreading throughout the body and accompanied by the sloughing of the epidermal mucus layer (9). Cases of GBD in *Andrias davidianus* present as erythema and congestion on the body surface, along with the formation of large numbers of gas bubbles in the major veins of the body cavity; histopathological examination reveals gas embolism-like lesions in the blood vessels of multiple organs (10).

In this study, gas bubbles were observed on the body surface, on the head, and within the abdominal cavity of affected *H. amjiensis* larvae, accompanied by symptoms such as abnormal buoyancy and decreased locomotor activity. These findings closely match the typical characteristics of GBD, further confirming the diagnosis. Although GBD is to some extent reversible, cases of animal mortality resulting from microvascular gas embolism and tissue damage are common (35, 36). Currently, it remains inconclusive whether GBD directly causes mortality in *H. amjiensis*, but for its larvae, which exhibit cannibalistic behavior, the disease creates a lethal vulnerability. Due to their sluggish movement and exposure while floating, affected individuals face a significantly increased risk of being preyed upon by conspecifics or natural predators, resulting in a sharp decline in survival rates. Consequently, GBD and the secondary risks it induces pose a serious threat to the early survival of this endangered species and may substantially reduce the success rate of larval metamorphosis and terrestrial emergence.

### 4.2 Environmental drivers contributing to gas bubble disease in *H. amjiensis*

Previous studies have shown that the supersaturation of TDG in water may be a direct cause of this disease (37). Under normal conditions, gases exist in a dissolved state in water and within organisms. However, when water is in a saturated or supersaturated state, an increase in temperature or a decrease in pressure can reduce gas solubility, causing gases to precipitate and form bubbles or gas emboli inside and outside the animal’s body, thereby inducing GBD (4, 38). When TDG levels are excessively high, particularly when dissolved oxygen increases significantly, it can lead to disease in fish and amphibians, and in severe cases, even death (39). The clinical symptoms observed in this study closely resemble those of known GBD in aquatic animals, suggesting that TDG supersaturation may also be a key trigger for GBD in *H. amjiensis*. In the natural shallow pools inhabited by *H. amjiensis*, the formation of TDG supersaturation is primarily driven by two factors: on the one hand, under conditions of ample sunlight, the photosynthesis of large quantities of algae promotes the rapid accumulation of oxygen in the water (40); on the other hand, the abundant organic matter in the bottom sediments undergoes reduction reactions in a relatively anoxic environment, continuously releasing nitrogen gas into the water (41, 42). More critically, the significant diurnal temperature fluctuations in mountainous areas cause the release of dissolved gases produced by photosynthesis during the day to slow as water temperatures drop at night, while the nitrogen-producing process in the bottom sediments is largely unaffected by the decrease in surface water temperature and continues to introduce gases into the water column (43). This cumulative effect of “daytime production and nighttime accumulation” may keep the water in a state of gas supersaturation for extended periods. It is therefore hypothesized that when *H. amjiensis* larvae breathe through their external gills and skin, the mixed gas permeating their bodies may be driven by pressure gradients to rapidly expand and precipitate in interstitial spaces and visceral veins, ultimately forming gas emboli visible to the naked eye.

In addition to the supersaturation of total dissolved gases, the concentrations of ammonia nitrogen and phosphate in the affected pools were significantly elevated, and the pH was markedly reduced, indicating a clear nutritional imbalance in the water. Although these factors are not direct causative agents of GBD, they can indirectly increase the risk of disease onset or even cause irreversible organ or tissue damage by altering the physicochemical environment of the water and the physiological state of the organism. In aquatic ecotoxicology, the toxicity of ammonia nitrogen is strictly regulated by pH. Although the vast majority of total ammonia nitrogen exists in ionic form under low pH conditions, thereby mitigating the central nervous system toxicity of molecular ammonia, when the pH falls below 4.8, the acidity itself can cause severe damage to the organism (44–46). Studies have shown that excessively low water pH can damage gill structure, reduce blood oxygen-carrying capacity, and slow metabolism, thereby increasing susceptibility to GBD (44). At the same time, elevated phosphorus levels can significantly promote explosive blooms of algae and cyanobacteria (47), further exacerbating abnormalities in the primary productivity of the water body. High concentrations of ammonia nitrogen can damage gill epithelial structures and impair respiratory and osmotic regulatory functions, making aquatic animals more susceptible to the effects of gas supersaturation (46), and may delay bubble dissipation by exacerbating metabolic disorders (45).

Analysis of meteorological data further indicates a high risk of GBD. Recently, the habitat of *H. amjiensis* has exhibited a trend of warmer winters and reduced precipitation (Figure 3), which has further decreased water exchange rates and promoted the accumulation of nutrients and algae. Meanwhile, rising spring temperatures and increased precipitation can transport humus and nutrients from surrounding leaf litter and dead vegetation into low-lying pools; the abundance of nutrients accelerates algal growth, contributing to the formation of gas supersaturation (Supplementary Figure S2). This water quality characteristic is consistent with the mild eutrophication reported by Yu et al. (23) in the breeding pools of *H. amjiensis* at Longwang Mountain. Therefore, the occurrence of GBD in *H. amjiensis* larvae may be the result of the combined effects of multiple factors, including light, temperature, nutrient enrichment, and precipitation.

### 4.3 Secondary intestinal dysbiosis induced by gas bubble disease

Previous studies have shown that GBD not only causes macroscopic physical damage but can also trigger systemic physiological dysfunction by affecting blood gas exchange and metabolic homeostasis (34). In this study, compared with healthy individuals, the fecal microbial community composition of diseased *H. amjiensis* larvae underwent a distinct shift. Notably, in the GBD group, the relative abundance of Fusobacteriota increased substantially to establish dominance, while the original dominant symbiotic communities (such as Pseudomonadota) relatively decreased. Additionally, Enterobacterales-related taxa and opportunistic pathogen-related bacteria such as *Aeromonas* increased concurrently, suggesting an expansion of facultative anaerobic and environmentally derived microbiota in the gut of diseased individuals and that the intestinal microecology may have shifted from a state of homeostasis to dysbiosis. Members of Fusobacteriota typically possess a strong capacity for the anaerobic fermentation of proteins and amino acids (48). Their enrichment may reflect enhanced intestinal anaerobic metabolic processes and may be accompanied by an increase in nitrogenous metabolic byproducts such as ammonia, thereby disrupting the local microenvironmental homeostasis of the gut (49). Many members of Enterobacterales are facultative anaerobes, and their expansion is often considered to be associated with oxidative stress, inflammation-related microenvironmental changes, and gut microbiota dysbiosis (50–52). Meanwhile, *Aeromonas*, an opportunistic pathogen widely present in aquatic environments, is prone to enrichment during host stress or decreased intestinal barrier function and has been reported to be closely associated with various aquatic animal diseases and tissue damage (53). Therefore, the synergistic enrichment of these three bacterial taxa may reflect the environmental stress caused by habitat eutrophication and water quality degradation. It also suggests that, under the GBD condition, the host gut may have developed an abnormal microenvironment characterized by the coexistence of enhanced oxidative stress, impaired barrier function, and low-grade chronic inflammation, thereby increasing the risk of opportunistic pathogen colonization and secondary infections. Such a microbiota shift may result from both the ingestion of more exogenous bacteria due to the diseased individuals’ exposure to deteriorating water quality and the selective enrichment of specific bacteria driven by internal intestinal environmental changes, which together ultimately lead to microecological dysbiosis.

Although it is currently difficult to establish a direct causal relationship between GBD and changes in the gut microbiota, their concurrent occurrence suggests that physiological damage caused by gas supersaturation may indirectly drive an imbalance in the gut microbial community structure by altering the intestinal microenvironment (e.g., redox status and barrier function). The decline in the abundance of dominant symbiotic bacteria observed in this study is similar to previous findings in other aquatic animals (e.g., *Pampus argenteus* and bullfrog tadpoles) (14, 15), which to some extent supports the aforementioned speculation. In addition, alterations in the physicochemical properties of aquatic environments may constitute important external factors that shape the gut microbiota of aquatic organisms (54). Previous studies have shown that acute or chronic ammonia-nitrogen stress can damage the intestinal mucosal structure of aquatic animals, including *Pelteobagrus fulvidraco* (55, 56) and *Penaeus monodon* (57), impair intestinal barrier function, and induce gut microbiota dysbiosis accompanied by the enrichment of potentially pathogenic bacteria (58). Ammonia-nitrogen stress may also disturb intestinal energy metabolic homeostasis by affecting the expression of genes involved in glucose metabolism, the tricarboxylic acid cycle, and the mitochondrial respiratory chain (59). Although direct evidence that elevated phosphate concentrations in water alter the structure or function of the host gut microbiota remains lacking, long-term exposure to high phosphate levels may induce oxidative stress, tissue damage, and metabolic abnormalities (60, 61), thereby indirectly affecting gut microbial communities through changes in the host’s physiological status. In the present study, ammonia-nitrogen and phosphate concentrations in the GBD group were markedly higher than those in the CON group and exceeded normal levels. These findings suggest that the gut microbiota alterations observed in the GBD group may also be associated with abnormalities in water physicochemical parameters.

Notably, in addition to external environmental factors, the host’s developmental stage, physiological condition, and feeding status may also shape the composition and structure of the gut microbiota (54). In the present study, diseased individuals generally exhibited reduced food intake or transient anorexia, which may have limited the availability of nutritional substrates in the gut and altered host energy metabolism, thereby further exacerbating gut microbial dysbiosis (62, 63). Moreover, owing to the limited sample size and the small quantity of feces obtainable from each individual, each sequencing sample in the GBD group consisted of feces pooled longitudinally from the same individual across different physiological stages, including the active phase of gas bubble disease, symptom recovery, and the resumption of feeding. This sampling strategy may partly explain why no significant differences were detected in *α*-diversity, whereas the GBD samples exhibited greater dispersion than the CON samples in the PCoA analysis. Taken together, the co-occurrence of gas bubble disease, gut microbial dysbiosis, and altered host physiology suggests that *H. amjiensis* larvae inhabiting pools with abnormal water quality may be exposed to the combined effects of multiple environmental and physiological stressors.

### 4.4 Compound ecological risks and recommendations for habitat conservation

GBD, intestinal microbial dysbiosis, and the resulting increased risk of cannibalism collectively indicate that *H. amjiensis* larvae are facing multiple stresses caused by deteriorating aquatic conditions. Therefore, it is imperative to implement scientific interventions to improve their breeding habitats. Regarding *in situ* conservation, it is recommended to install small-scale barriers around the core breeding pools to intercept external organic matter—such as leaf litter—carried by surface runoff, thereby reducing the input of organic matter that triggers bottom sediment oxygen depletion and eutrophication at the source. Concurrently, native wetland vegetation around the pools should be moderately restored to utilize the plants’ absorption capacity and reduce the nutrient load in the water. Furthermore, introducing natural water sources to promote water exchange can be attempted; this not only helps to dilute abnormal physicochemical parameters but also accelerates the dissipation of supersaturated gases through physical disturbance. For pools with severely degraded water quality, excessive algal blooms, and high larval morbidity rates, appropriate interventions can be implemented prior to breeding, such as adding probiotics to accelerate the decomposition of humus at the pool bottom, reducing ammonia nitrogen production, preventing nutrient enrichment that triggers algal blooms, and moderately increasing water volume. If cases of GBD are detected during the larval stage, preventive temporary rearing and rescue measures should be implemented promptly to reduce the risk of predation and mortality among the affected individuals. In summary, strengthening long-term monitoring of the breeding water environment, implementing targeted habitat restoration, and employing scientific rescue measures are crucial steps. These strategies may be among the effective ways to reduce the risk of early larval mortality and improve the success rate of terrestrial emergence for *H. amjiensis*.

## 5 Conclusion

This study is the first to document the clinical symptoms of gas bubble disease in wild *H. amjiensis* larvae, and it analyzes the potential factors contributing to the disease and its secondary effects on the host. Eutrophication and gas supersaturation in the habitat pools are likely the key environmental drivers of this disease. Furthermore, the disease not only causes physical lesions in the larvae but also triggers intestinal microecological dysbiosis characterized by the loss of core symbiotic bacteria and the accumulation of opportunistic pathogens. This dual “environmental-physiological” stress increases the risk of mortality for *H. amjiensis* larvae. Therefore, we recommend that conservation authorities incorporate long-term water quality monitoring of breeding pools and *in situ* habitat restoration into their long-term conservation management efforts. This will help mitigate the environmental stress caused by rising temperatures and reduced precipitation in recent years, improve the success rate of larval terrestrial emergence, and ensure the stable development of the wild population of this critically endangered amphibian.

## Data Availability

The data presented in the study are deposited in the NCBI Sequence Read Archive (SRA) repository, accession number PRJNA1513654.
