# Supplementary material for: Clinical observations, environmental parameters, and gut microbiota alterations associated with gas bubble disease in the endangered Anji salamander (Hynobius amjiensis)
Source: Front Vet Sci. 2026 Aug 28;13:1900113. doi: 10.3389/fvets.2026.1900113 (PMC13564460; doi:10.3389/fvets.2026.1900113)
Supplement: Supplementary file 1 [file Table_1.DOCX]

Supplementary Material

# 1 Supplementary Figures

**Supplementary Figure 1.** **Macroscopic clinical manifestation of gas bubble disease (GBD) in a wild *Hynobius amjiensis* larva.** (A) An overall view of the affected larva temporarily placed in a transparent container for examination. Due to the incidental nature of the field encounter, the image was captured opportunistically using a mobile device immediately upon observation. The red box highlights the primary focal area of the macroscopic lesion on the cranial region. (B) A magnified view of the head region indicated by the red square in panel A. The red arrow points to a distinct, large, and transparent subcutaneous gas bubble located anterior to the eye, near the snout/rostrum.

**Supplementary Figure 2.** **Environmental conditions of the natural breeding habitat of the critically endangered *Hynobius amjiensis*.** (A) The breeding habitat during the spring season (April to May). The natural Sphagnum bog is visibly overgrown and covered by abundant competitive weeds and other vegetation. (B) Interspecific competition between other vegetation and *Sphagnum* moss occurs within the *H. amjiensis* habitat. The red arrow (labeled B1) points to the native *Sphagnum* moss, a critical substrate in the breeding habitat of *H. amjiensis*. However, the aggressive overgrowth of surrounding weeds creates severe resource competition with the *Sphagnum* moss, which may lead to the shrinkage and degradation of this crucial microhabitat. (C) A breeding pool during the severe winter. Extremely low ambient temperatures cause the breeding pools of *H. amjiensis* to freeze completely, creating a harsh overwintering environment. (D) A breeding pool following spring warming and ice melt. Although the ice layer has melted, the water level remains critically low, indicating a severe scarcity of water resources required for early larval development. (E) A different view of a breeding pool in the spring when it is getting warmer. In addition to the persistently low water levels, the accumulation of organic matter and water quality deterioration can be observed in some breeding pools, which may further act as environmental stressors for the developing larvae.

# 2 Supplementary Tables

**Supplementary Table 1. Clinical observations, feeding events, and recovery records of *Hynobius amjiensis* larvae during the temporary observation period.**

| **Individual ID** | **Bubble distribution** | **Body weight (g)** | **Total length (mm)** | **Time to bubble disappearance(h)** | **Total number of feeding events** | **Total number of defecation events** |
| --- | --- | --- | --- | --- | --- | --- |
| GBD1 | Snout region | 0.65 | 42.00 | 24 | 5 | 6 |
| GBD2 | Subcutaneous trunk | 0.72 | 46.00 | 24 | 5 | 5 |
| GBD3 | Abdominal cavity | 0.78 | 48.00 | 48 | 4 | 4 |
| GBD4 | Right posterior abdomen | 1.18 | 52.00 | 72 | 2 | 2 |
| CON1 | No visible gas bubbles | 1.38 | 55.00 | NA | 7 | 7 |
| CON2 | No visible gas bubbles | 1.09 | 49.00 | NA | 7 | 6 |
| CON3 | No visible gas bubbles | 0.76 | 45.00 | NA | 7 | 5 |
| CON4 | No visible gas bubbles | 1.28 | 51.00 | NA | 7 | 5 |

Note: GBD, larvae with suspected gas bubble disease; CON, control larvae without visible gas bubbles. Clinical observations were conducted during a 7-day temporary observation period. Bubble distribution was recorded based on visible gas bubbles observed on the body surface or in the abdominal region. Time to bubble disappearance was defined as the time from transfer to aerated, dissolved-gas-equilibrated water until visible bubbles were no longer observed. The total number of feeding events and the total number of defecation events represent the cumulative numbers recorded during the 7-day temporary observation period. NA, not applicable because no visible gas bubbles were observed in control larvae.

**Supplementary Table 2. Summary of sequencing quality and OTU clustering results**

| **Item** | **Value** |
| --- | --- |
| Amplified region | 338F_806R |
| Insert size | 468 bp |
| Sequencing length | PE300 |
| Number of samples | 6 |
| Raw reads per end | 335,291 |
| Total bases of raw single-end reads | 100,587,300 bp |
| High-quality merged sequences | 314,474 |
| Total bases of high-quality merged sequences | 130,302,524 bp |
| Average sequence length after processing | 414 bp |
| OTU clustering threshold | 97% sequence identity |
| Total clustered OTUs | 966 |

Note: Raw reads per end refers to the number of reads generated from one sequencing direction. For paired-end sequencing, the total number of raw single-end reads was calculated as twice the number of raw reads per end. High-quality merged sequences refer to sequences retained after paired-end read merging, quality filtering, and chimera removal. OTUs were clustered at 97% sequence similarity.

**Supplementary Table 3. Per-sample sequencing metrics after read merging and quality filtering**

| **Sample ID** | **Effective sequences** | **Base number (bp)** | **Mean length (bp)** | **Min length (bp)** | **Max length (bp)** |
| --- | --- | --- | --- | --- | --- |
| GBD1 | 63,690 | 26,001,121 | 408.24495 | 261 | 449 |
| GBD2 | 56,887 | 24,238,252 | 426.07717 | 262 | 432 |
| GBD3 | 66,784 | 27,385,765 | 410.06476 | 275 | 430 |
| CON1 | 42,589 | 17,692,107 | 415.41494 | 259 | 433 |
| CON2 | 41,629 | 17,209,368 | 413.39854 | 261 | 447 |
| CON3 | 42,895 | 17,775,911 | 414.4052 | 399 | 433 |
| Total | 314,474 | 130,302,524 | 414.6009267 | 259 | 449 |

Note: GBD, gas bubble disease group; CON, healthy control group.
